# Experimental Study for Damage Identification of Storage Tanks by Adding Virtual Masses

**DOI:** 10.3390/s19020220

**Published:** 2019-01-09

**Authors:** Jilin Hou, Pengfei Wang, Tianyu Jing, Łukasz Jankowski

**Affiliations:** 1 Department of Civil Engineering and State Key Laboratory of Coastal and Offshore Engineering, Dalian University of Technology, Dalian 116023, China; wang_pengfei@mail.dlut.edu.cn (P.W.); jty6666@163.com (T.J.); 2 Key Laboratory of Structures Dynamic Behavior and Control, Ministry of Education, Harbin Institute of Technology, Harbin 150090, China; 3 Institute of Fundamental Technological Research, Polish Academy of Sciences, 02-106 Warsaw, Poland; ljank@ippt.pan.pl

**Keywords:** damage identification, storage tanks, sensitivity analysis, frequency

## Abstract

This research proposes a damage identification approach for storage tanks that is based on adding virtual masses. First, the frequency response function of a structure with additional virtual masses is deduced based on the Virtual Distortion Method (VDM). Subsequently, a Finite Element (FE) model of a storage tank is established to verify the proposed method; the relation between the added virtual masses and the sensitivity of the virtual structure is analyzed to determine the optimal mass and the corresponding frequency with the highest sensitivity with respect to potential damages. Thereupon, the damage can be localized and quantified by comparing the damage factors of substructures. Finally, an experimental study is conducted on a storage tank. The results confirm that the proposed method is feasible and practical, and that it can be applied for damage identification of storage tanks.

## 1. Introduction

With recent advances in structure health monitoring [1,2,3,4,5] and damage detection [6,7,8,9,10], new technologies and methods has been proposed and developed. With the development of petrochemical industry and the implementation of the National Petroleum Strategic Reserve Plan in China, more and more tanks will be in construction and service. However, once accidents happen to these tanks, they might result in loss of life and property. Therefore, the problem of monitoring of the tanks that are in service has attracted more and more attention. Aiming at this problem, this paper proposes and verifies a practical and accurate method for the damage identification of storage tanks.

At present, the detection methods applicable to monitoring of tanks mainly include X-ray detection [11], acoustic emission [12,13,14], ultrasonic detection [15,16,17,18], magnetic flux leakage detection [19,20], eddy current [21], etc. The advantage of these nondestructive detection methods [22] is that they do not depend on the finite element model [23] of the structure; however, they cannot quantitatively evaluate the degree of damage. Some of them are also time-consuming and costly because of the need to empty the storage tanks prior to damage identification.

In recent years, damage identification techniques based on vibration testing [24,25] have been widely developed and applied; it is very important in such approaches to select a suitable damage index [26]. These methods can identify the damage before it is visible [27,28], so that the structure can be repaired before the damage becomes severe. Structural modes are characteristic functions of physical parameters of structures [29], so that changes of physical parameters inevitably lead to changes of structural modes [30]. The modes are thus widely used in structural damage identification [31]. Methods that are based on modal analysis and frequency response are widely used in damage identification of civil engineering structures like bridges or cable structures, and their feasibility in such cases has been validated in practice. However, actual storage tanks are cylindrical and usually circumferentially symmetrical, so that various similar damages at different locations (at the same height) may result in exactly the same modal change. Such problems increase the difficulty of damage identification of tank structures and limit the scope of application of the modal-based methods.

Recently, the method of additional masses, based on structural modes and frequencies, has been applied in damage identification. Kołakowski et al. [32] use the Virtual Distortion Method (VDM) to identify structural damage both in static and in dynamic analysis, and the results are verified by experiments and numerical simulations. Kołakowski et al. [33] demonstrate the capability of VDM in linear and nonlinear analysis and proves it to be an efficient reanalysis tool in various applications. Dackermann et al. [34] add physical masses to a two-story framed structure to stimulate frequency changes due to structure damage. Based on this methodology, Suwala et al. [35] present a model-free method for off-line identification of structural mass modifications and verify it experimentally. Lu et al. [36] add masses to various beam structures and analyze their influence on the process of structural damage identification; it is concluded that the value, number, and position of additional masses all have significant effect on the accuracy of damage identification. Adding masses to a structure can greatly increase the quantity of modal information and its sensitivity to damage [37], but it is usually difficult and inconvenient to add real masses to structures.

In 2018, Hou et al. [38] add virtual masses to a plane frame structure by the VDM, and the effectiveness of the method is validated by numerical simulation and in experiment. In this paper, the idea of using additional masses to increase the amount and sensitivity of the test data is used to improve the accuracy of damage identification of tank structures. Furthermore, when combined with the basic idea of the VDM, the feasibility of the method is improved by employing virtual masses instead of actual physical masses. Based on the VDM, only one set of excitations and the corresponding accelerations are measured to construct numerically the frequency response of the structure with added virtual masses. In practical engineering applications, it avoids the significant difficulties of adding physical masses and reduces the difficulty of practical operation.

Firstly, this paper introduces how the VDM is applied to add virtual masses. Subsequently, the damage location is preliminarily estimated based on the obtained modal information of substructures with the additional virtual masses. Furthermore, the damages of the substructures with additional virtual masses are quantified based on the response sensitivity, and finally the effectiveness of the method is verified through the tank model.

## 2. Damage Identification of Storage Tanks Using Additional Virtual Masses

### 2.1. Adding Virtual Masses

Based on the basic idea of the VDM [18,22], the frequency response of the structure after adding the virtual mass m can be numerically constructed using a set of excitations and the corresponding acceleration responses. The added mass is called virtual, because the process is purely numerical and it involves no physical masses. The formula is as follows:

(1) $$H_{pp}\left(\omega ,m\right)=\frac{A\left(\omega \right)}{F\left(\omega \right)+mA\left(\omega \right)}$$

where $H_{pp}\left(\omega ,m\right)$ represents the acceleration frequency response after adding the virtual mass m to the direction of the degree of freedom *p*. F(ω) and A(ω) represent the spectra of the measured excitation forces and the accelerations in the direction of the degree of freedom *p*, respectively. The mass m is the additional virtual mass. Adding a virtual mass affects the natural frequencies of the structure and their sensitivity with respect to damage. Specific values of the added masses are selected to maximize these sensitivities, and the resulting modified, sensitive natural frequencies are used for damage identification.

### 2.2. Preliminary Localization of Damage

General storage tanks are often cylindrical with the wall thickness depending on the height only (constant in the circumferential direction). Based on this structural characteristic, the location and degree of damage can be preliminarily estimated by analyzing the frequencies of the structure with a mass sequentially added in consecutive points of the circumferential direction.

**Figure 1:**
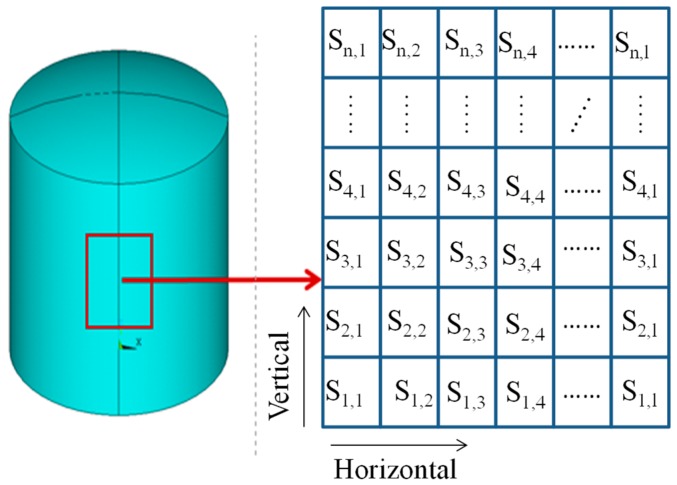
Schematic sketch of substructures.

An area of storage tanks is selected to be identified; it can be either the entire tank or a local area. The region is divided into *n* × *l* substructures, which are denoted by $S_{V,H}$ (Figure 1). The subscript *V* represents the index in the vertical direction and the subscript H represents the index in the circumferential/horizontal direction, where *V* =1,2,…n, H=1,2,…l. The same virtual mass $m_{V}$ is sequentially added to each substructure in a loop and the obtained (virtual) new structure is denoted by $G_{V,H}\left(\mu ,m\right)$. The first natural frequency with a high sensitivity is expressed by ${\tilde{\omega }}_{V,H}\left(m_{V}\right)$.

If there is no damage in the structure, then the dynamic characteristics of all the substructures $G_{V,H}\left(\mu ,m\right)$ are the same when the height *V* is fixed, and so also ${\tilde{\omega }}_{V,H}\left(m_{V}\right)$ does not depend on *H*. Therefore, by inspecting the frequency ${\tilde{\omega }}_{V,H}\left(m_{V}\right)$ in the circumferential direction *H*, the location and the extent of the damage can be preliminarily determined.

Along the direction of height (indexed with the subscript *V*), the frequency ${\tilde{\omega }}_{V,H}\left(m_{V}\right)$ varies even without damage, because the distance between the substructures and the boundaries (as well as the wall thickness) are different. In order to facilitate the comparison of the frequencies, they are first normalized with respect to the maximum frequency at each height *V*. The damage identification factor $D_{V,H}$ is introduced in Equation (2), where ${\tilde{\omega }}_{V\max}$ is the maximum frequency in the row *V*. By assessing the values of $D_{V,H}$, the damage of each substructure in the considered region can be qualitatively estimated.

(2) $$D_{V,H}=1-\frac{{\tilde{\omega }}_{V,H}}{{\tilde{\omega }}_{Vmax}}$$

(3) $${\tilde{\omega }}_{Vmax}=max\left({\tilde{\omega }}_{V,1},{\tilde{\omega }}_{V,2},\ldots ,{\tilde{\omega }}_{V,l}\right)$$

### 2.3. Damage Identification Based on Sensitivity

If the above method is used to determine whether there is damage in the tank, the damage extent can be further quantitatively identified by employing a finite element model.

The natural frequencies of the virtual structures that were obtained in the previous section are called experimental frequencies, and the counterpart frequencies of the finite element model of the tank with added masses are called numerical frequencies. The total square error between the numerical and the experimental frequencies is taken as the objective function to be minimized by optimizing the substructure parameters, so that the damage is quantified in this way. The objective function T(μ) is defined as in Equation (4). In order to express it conveniently, the analyzed natural frequencies of all the substructures are uniformly indexed by a common single index *i* (instead of two separate indices *V* and *H*). The Gauss–Newton method is adopted as the optimization method.

(4) $$T\left(\mu ,m\right)=\frac{1}{2}\overset{n}{\underset{i=1}{\sum }}{\left(∆\omega_{i}\left(\mu ,m\right)\right)}^{2}$$

The relative error $∆\omega_{i}\left(\mu ,m\right)$ between the numerical natural frequency $\omega_{i}\left(\mu ,m\right)$ and the experimental natural frequency $\omega_{A,i}\left(m\right)$ of the *i*-th substructure with the added virtual mass *m* is expressed as in Equation (5). The damage factor μ is the stiffness reduction ratio of the substructure before and after the damage, and its maximum is equal to 1.

(5) $$∆\omega_{i}\left(\mu ,m\right)=\frac{\omega_{A,i}\left(m\right)-\omega_{i}\left(\mu ,m\right)}{\omega_{A,i}\left(m\right)}$$

The first derivative and the second order derivative of T(μ) can be obtained:

(6) $$\nabla T\left(\mu \right)=-P^{T}\left(\mu \right)∆\omega \left(\mu ,m\right)$$

(7) $$\nabla^{2}T\left(\mu \right)\approx P^{T}\left(\mu \right)P\left(\mu \right)$$

where ∆ω(μ,m) is the vector that collects the relative errors of all substructures, and *P* is the Jacobi matrix of ∆ω(μ,m) with respect to μ:

(8) $$\{\begin{array}{c} \mu ={\left[\mu_{1},\mu_{2},\ldots ,\mu_{n}\right]}^{T}\\ ∆\omega ={\left[∆\omega_{1},∆\omega_{2},\ldots ,∆\omega_{n}\right]}^{T}\\ P=\left[P_{1}^{T},P_{2}^{T},\ldots ,P_{n}^{T}\right]\\ P_{i}^{T}={\left[\frac{\partial \omega_{i}\left(\mu_{1}\right)}{\partial \mu_{1}},\frac{\partial \omega_{i}\left(\mu_{2}\right)}{\partial \mu_{2}},\ldots ,\frac{\partial \omega_{i}\left(\mu_{n}\right)}{\partial \mu_{n}}\right]}^{T} \end{array}$$

As in the Newton method, the Taylor expansion of ∇T(μ) is performed at $\mu_{0}$ up to the second term, while the higher-order terms are neglected:

(9) $$\nabla T\left(\mu \right)\approx \nabla T\left(\mu_{0}\right)+\nabla^{2}T\left(\mu_{0}\right)(\mu -\mu_{0})$$

To obtain the minimum value of T(μ), the first derivative of T(μ), i.e., ∇T(μ) is supposed to be zero, which yields the following update formula that should lead towards the extreme point of T(μ):

(10) $$\mu =\mu_{0}-(\nabla^{2}T\left(\mu_{0}\right){)}^{-1}\nabla T\left(\mu_{0}\right)$$

Equations (6) and (7) are substituted into Equation (10) to obtain Equation (11).

(11) $$\mu =\mu_{0}+P^{+}∆h\left(\mu ,m\right)$$

The matrix $P^{+}$ represents the generalized inverse matrix of P, which can be obtained by the singular value decomposition (SVD). The damage factor μ can be approximated by Equation (11). Subsequently, the obtained μ is taken as the initial value of the next iteration and a new sensitivity matrix *P* is obtained by using the new structural parameters. The above steps are repeated until the iterations converge.

## 3. Experiments of Tanks

### 3.1. Models of the Experiment

**Figure 2:**
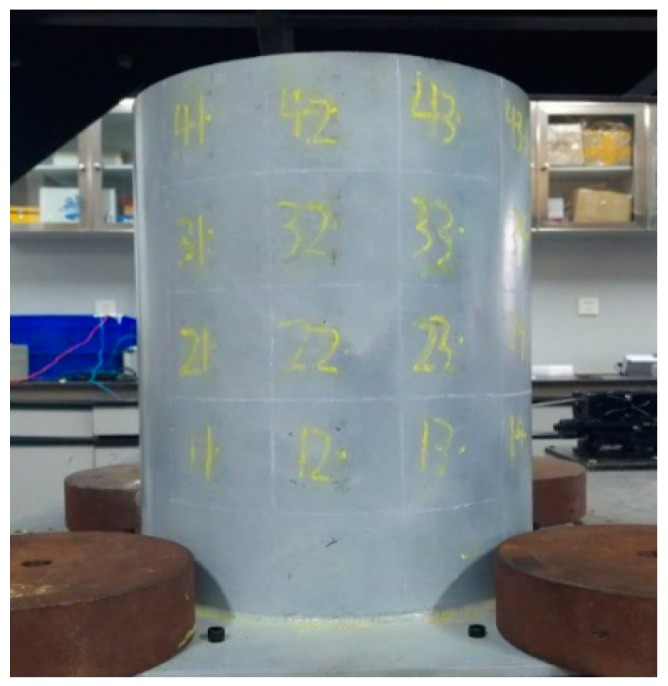
The damage area to be identified.

The model in the experiment is a small cylindrical storage tank without a cap, as shown in Figure 2. For the tank model, the diameter of the tank wall is 300 mm. The height is 400 mm and the thickness of the side wall is 2 mm. The elastic modulus of steel structure of the tank is assumed to be 206 GPa and the density is 7.85 × 10^−3^ g/mm^3^. The tank wall is welded to a rectangular steel plate and the size of the bottom plate is 370 mm × 370 mm with a thickness of 8 mm. The tank model is bolted to the optical test platform, and a counterweight block is placed at each corner of the tank bottom plate in order to ensure full contact between the tank bottom and the test platform.

**Figure 3:**
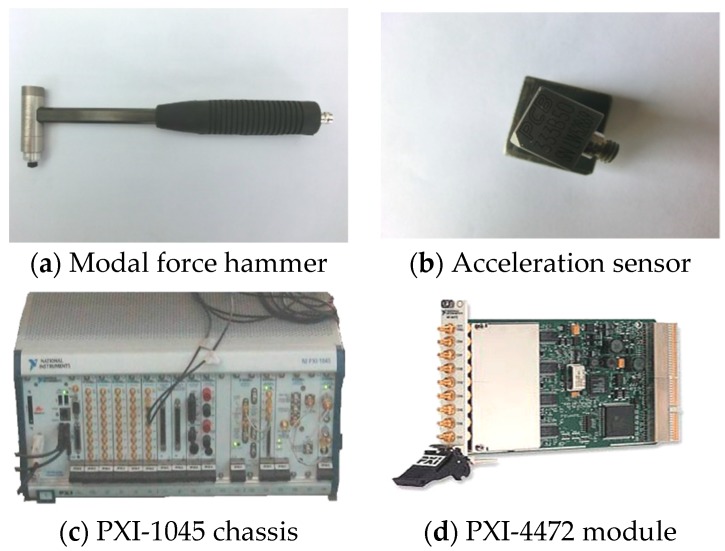
Instruments.

In this experiment, a force hammer is used to exert the excitation load. The force hammer is produced by PCB Company (New York City, NY, USA) and it is shown in Figure 3a. It is equipped with a force sensor that is capable of measuring the excitation force [39]. In order to avoid repeated rebound excitations, a hard rubber hammer head is selected in this experiment, so that a smooth and gentle exciting force can be obtained. Acceleration response acquisition sensor is a single-axis accelerometer that is produced by PBC Company (USA) and is shown in Figure 3b. It is difficult to connect the accelerometer with the tank model because the side wall of the tank structure is a smooth circular steel plate and the liquid needs to be stored in it. In this experiment, a high-strength magnet was installed at the bottom of the sensor and fixed with screws, so that the sensor can be easily and quickly installed and removed. The total weight of the sensor and magnet is 27.7 g.

The signal acquisition equipment is the PXI-1045 chassis and the dynamic signal acquisition module NIPXI-4472 produced by National Instrument (NI), as shown, respectively, in Figure 3c,d. The PXI-1045 supports the LabVIEW software, which is used to collect data according to the acquisition needs. The PXI-4472 acquisition module has eight channels, which is enough to collect the exciting force signal of the hammer and the acceleration response signal of the acceleration sensor.

### 3.2. Numerical Finite Element Model

#### 3.2.1. Foundation of the Model

The finite element model of storage tank is established in ANSYS and the shell63 element is adopted for both the tank wall and the bottom plate. In grid generation, the tank wall is divided into 280 elements, and the tank bottom is divided into 108 elements, totaling 388 elements. Each substructure of the tank wall is composed of four elements.

**Figure 4:**
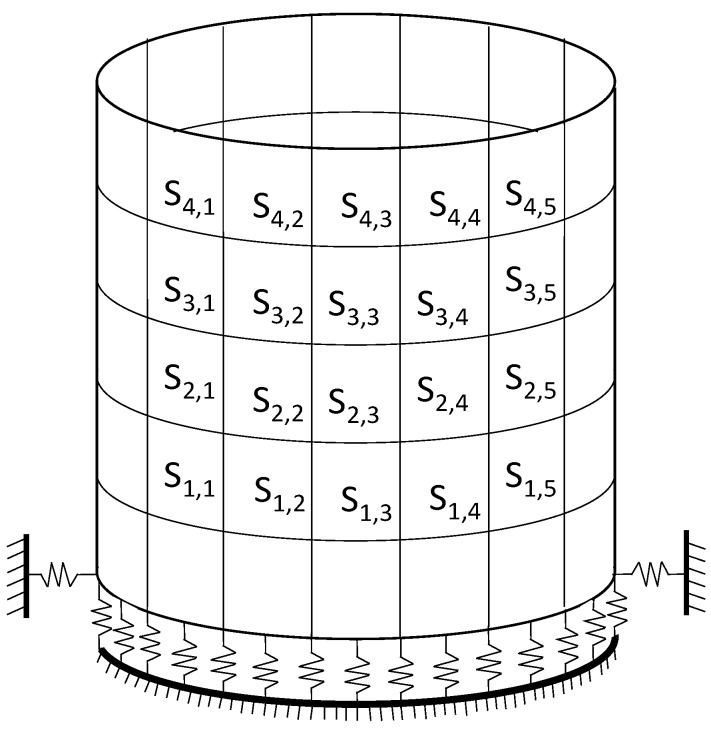
Structure of the storage tank model: substructures and spring supports.

A vertical three-dimensional spring element combin14 was added under each node of the tank floor to simulate the effect of the test platform on the upper structure of the tank, and a horizontal toroidal three-dimensional spring element combin14 was set to simulate the effect of bolts and counterweight blocks on the tank. The vertical spring stiffness is 5 × 10^6^ N/m and the horizontal spring stiffness is 6 × 10^7^ N/m. Schematic sketch of the spring support is shown in Figure 4.

#### 3.2.2. Modal Analysis

**Figure 5:**
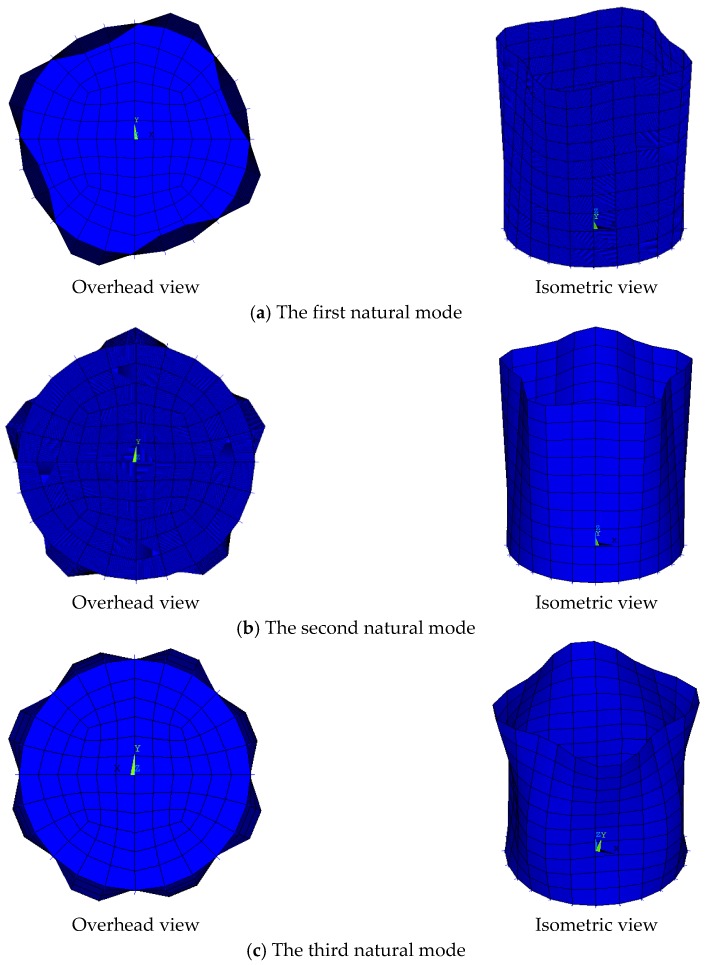
The first three natural modes of the tank model.

The first three natural frequencies of the tank model (Table 1) and the first three non-repeating natural vibration modes (Figure 5) are obtained by ANSYS. Because the tank structure is a symmetrical spatial structure, there are repeated frequencies, and several frequencies are very similar. In order to facilitate the optimization and calculation of structural parameters, the stiffness matrix and the mass matrix are derived by ANSYS, and the subsequent calculations are carried out by MATLAB.

#### 3.2.3. Model Modification

The elastic modulus of steel is given in the FE model, but the assumed value is uncertain due to different kinds of steel that could be used for the tank. Therefore, the assumed elastic modulus of tank steel is corrected, and the correction factor *K* is defined as the ratio of the actual elastic modulus *E* of the tank structure to the numerical value assumed in the FE model. The proper value of the correction factor *K* is found by comparing the first two natural frequencies of the tank with an added virtual mass (as calculated by the finite element model established in ANSYS and MATLAB) with the corresponding frequencies that were found experimentally with real physical mass added to the tank structure. By minimizing the sum of the squares of the frequency differences, the optimum *K* is found to be 0.9223 and so the revised elastic modulus is 190 GPa. The comparison between the frequencies before and after modification and the experimental values is shown in Table 2.

#### 3.2.4. Partition into Substructures

One-third of the tank wall is selected as the area to be identified. Because the steel plate at the bottom of the tank wall is close to the weld and the stiffness error is large, it is not studied. Accordingly, the region to be identified is divided into 20 substructures, denoted by *S_V,H_*, where *V* and *H* represent the vertical and horizontal positions of the substructures in the original structure respectively.

#### 3.2.5. Construction of Virtual Masses

**Figure 6:**
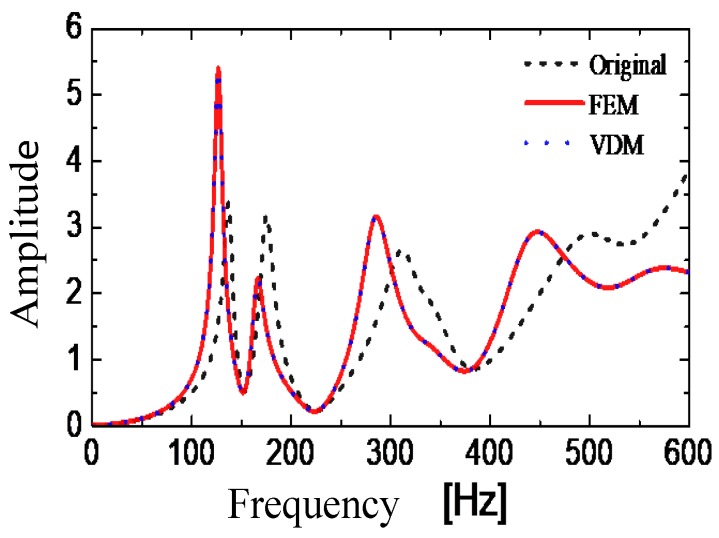
Frequency response of the undamaged tank: without added mass (Original), and with an additional mass, as constructed by the FEM (Finite Element Method) and by the Virtual Distortion Method (VDM).

The acceleration frequency response is obtained by applying the excitation perpendicularly to the middle of the wall of the substructure *S*_2,3_ in the numerical undamaged model. The amplitude of the acceleration frequency response is shown in Figure 5 (Original). Subsequently, 0.6 kg of mass is added to the substructure in the numerical model and the amplitude of the resulting acceleration frequency response is shown in Figure 5 (FEM, Finite Element Method). Finally, the amplitude of the frequency response after adding 0.6 kg of virtual mass is calculated by the VDM by substituting the original frequency response into Equation (1), and the result is shown in Figure 6 (VDM). The curves VDM and FEM can be compared to be identical. It shows that the VDM accurately computes the frequency response of the structure with added virtual masses, so that one does not need to assemble a finite element model for this purpose, which greatly improves the calculation efficiency due to the simplicity of Equation (1).

In addition, it can be seen from Figure 5 that the excitation of the original tank results in several low-order modes being excited with comparable amplitudes. However, after adding the virtual mass, the tank vibrates mainly in its first natural mode, which is easier to be determined and used for damage identification. The subsequent sensitivity analysis shows that the mode has a high sensitivity to damage.

#### 3.2.6. The Relation between Sensitivity and Added Masses

**Figure 7:**
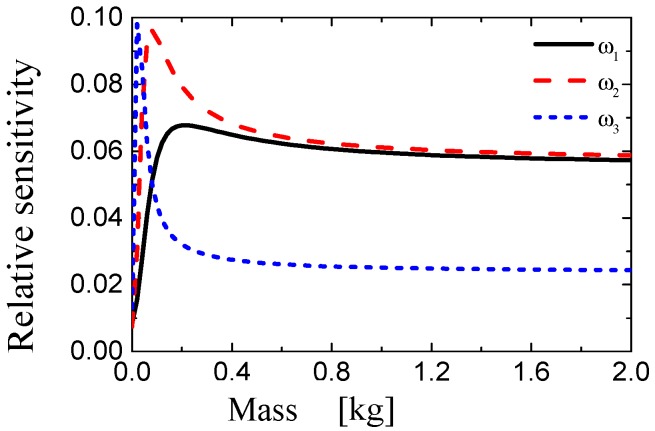
The relation between relative sensitivity and additional masses after adding virtual masses.

First, the virtual mass *m* is added to the middle of the substructure *S*_3,3_. The relative sensitivity of the first three frequencies of substructure *S*_3,3_ is calculated and shown in Figure 7. The formula for calculating the relative sensitivity is shown in Equation (12).

(12) $$R_{lp}=\frac{\partial \omega_{ip}}{\omega_{p}\partial \mu_{l}}=\frac{\varphi_{p}^{T}K_{l}\varphi_{p}}{2\omega_{p}^{2}}$$

The relative sensitivity is the sensitivity normalized with respect to frequency, which facilitates the comparison of the sensitivities of several natural frequencies.

As can be seen from Figure 7, the relative sensitivity of the first natural frequency increases rapidly with the increase of the mass value, reaches its maximum at 0.2 kg, and then decreases slowly. When considering the difference between the numerical model and experimental model, we finally decide to use 0.3 kg as the minimum value of added mass, because when the mass value exceeds 0.3 kg, the relative sensitivity of the first natural frequency is relatively high and stable. The relative sensitivity of the second natural frequency achieves its maximum at 0.1 kg, and then decreases slowly. Finally, the relative sensitivity of the third natural frequency reaches its maximum initially value very quickly, and then drops quickly, too. Virtual mass is then sequentially added to the other 19 substructures to construct a full set of 20 virtual structures $G_{V,H}\left(\mu ,m\right)$. For damage identification purposes, the first natural frequency is selected by analyzing the relationship between the sensitivity and the added virtual masses of all 20 substructures.

### 3.3. Verification of Virtual Masses Method

**Figure 8:**
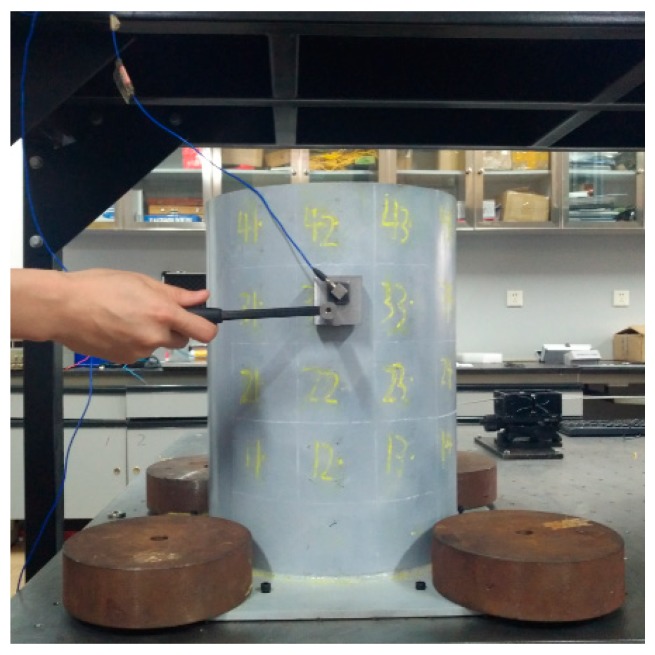
The tank model.

**Figure 9:**
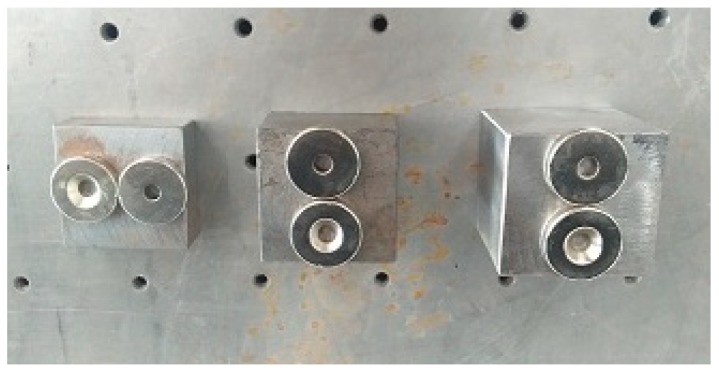
Masses.

**Figure 10:**
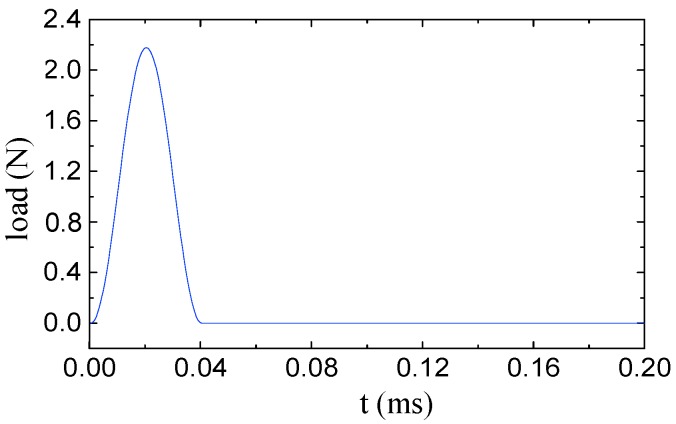
The excitation force.

**Figure 11:**
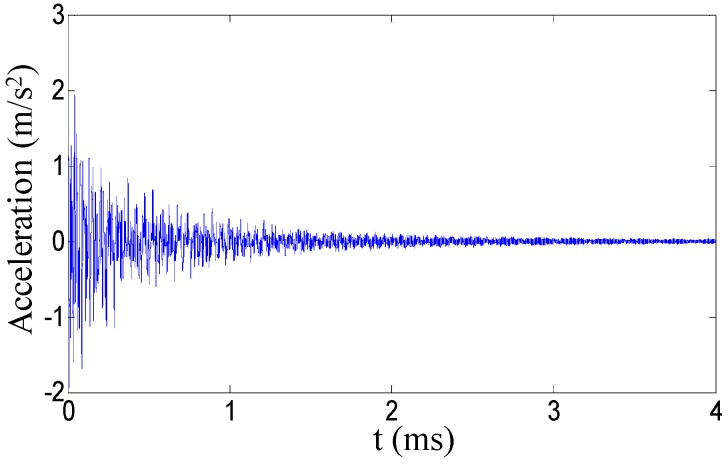
The response.

**Figure 12:**
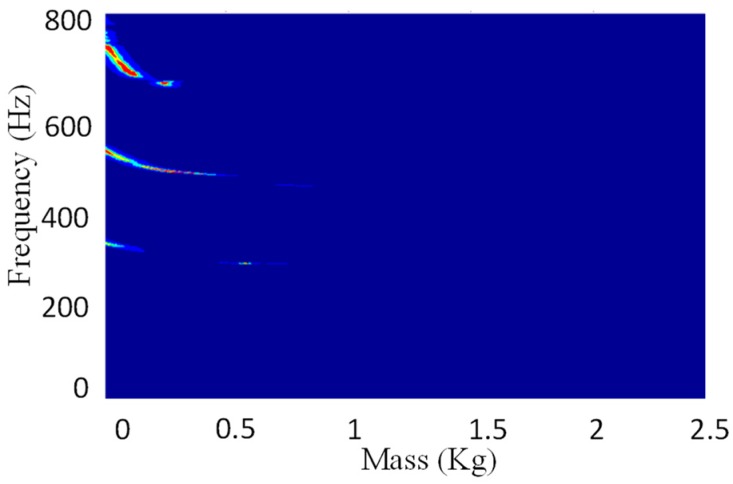
Contour plot of the experimental model of the substructure after adding virtual mass.

For the tank test model (Figure 8), an additional verification of the procedure for virtual mass addition is performed. The second natural frequency of the four substructures in the column *V* = 2 is taken as an example. In the middle of each substructure, the modal force hammer is used to excite the tank in the direction perpendicular to the wall. The acceleration response in the corresponding position is collected by the acceleration sensor. The frequency response with the added virtual mass is constructed by Equation (1), and the second natural frequency is extracted for additional 0.3 kg, 0.6 kg and 0.9 kg virtual masses while using the peak value extraction method. Subsequently, the actual mass blocks of 0.3 kg, 0.6 kg, and 0.9 kg shown in the Figure 9 are added in the middle of each substructure. After applying the excitation (Figure 10), the acceleration response (Figure 11) at the corresponding position is measured and the corresponding frequency response (with added real mass) is obtained by the FFT algorithm. The contour plot of the experimental model of the substructure after adding virtual mass is shown in Figure 12. The brightness reflects the amplitude of the frequency response, that is to the natural frequencies of the virtual structure. Comparing the second natural frequency of the substructures *V* = 2 with real mass and with virtual mass, it can be found in Table 3 that the frequency of the substructure with real mass is very close to that computed by the VDM, and the error is very small.

### 3.4. Damage Identification of the Damaged Storage Tank

**Figure 13:**
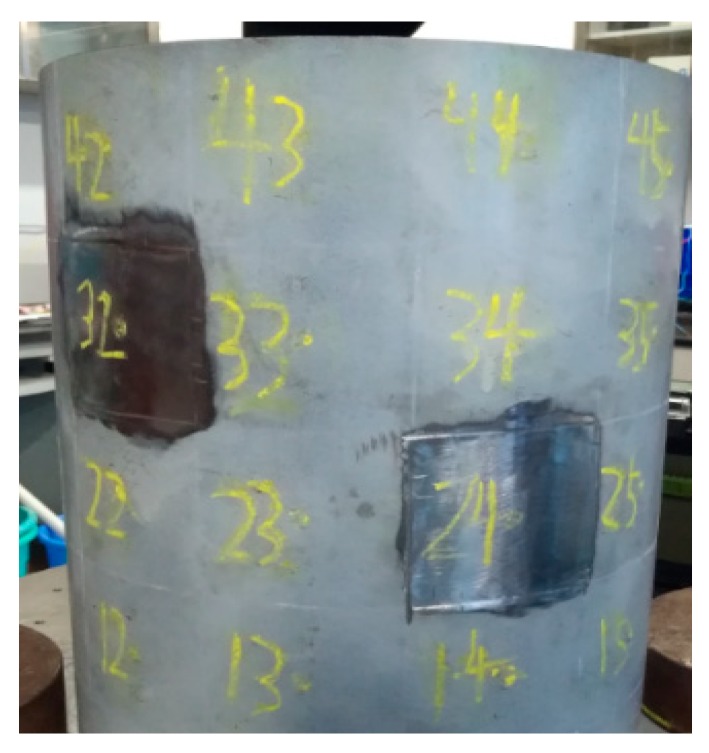
The damage of tank.

The validity of the VDM-based method for adding virtual masses presented in Section 2.1 is verified in an experiment of the original tank structure model without any additional damages. For the purpose of damage identification, the wall thicknesses of substructures *S*_3,2_ and *S*_2,4_ are reduced by 0.6 mm and 0.4 mm, respectively, by grinding, as shown in Figure 13. The damage location is then identified by comparing the dynamic information data of each substructure circumferentially. Finally, the damage factor of the substructure was obtained by sensitivity iteration coupled with the numerical FE model.

#### 3.4.1. The Comparison of Structural Frequency before and after Damage

**Figure 14:**
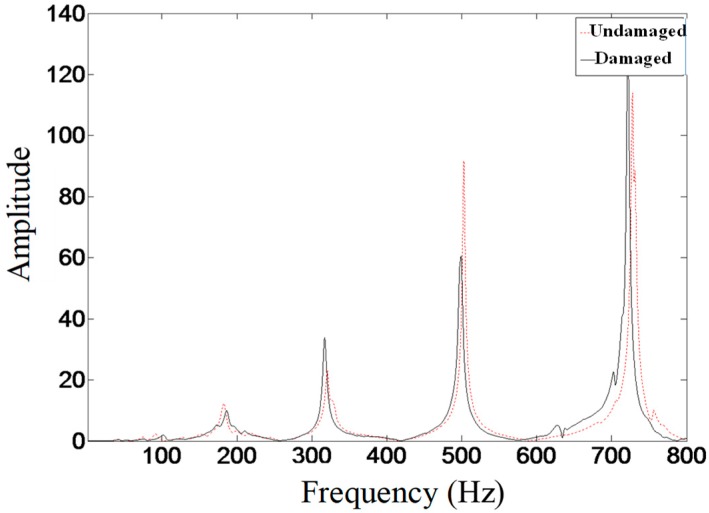
Frequency domain response of substructures before and after damage.

By measuring the dynamic response information of the tank structure before and after damage, the first two natural frequencies before and after damage are obtained. The excitation force is applied in the middle position of the substructure before and after the damage, and the acceleration frequency response is measured, as shown in Figure 14.

As can be seen from Table 4 and Figure 14, the difference of the frequency-domain response of the tank structure before and after damage is very small, and the frequency responses are very close. Therefore, such overall information of the structure is hardly useful as an indicator to identify the structural damage. It is necessary to combine the local response information of the structures to identify the damage.

#### 3.4.2. Localization of Damage

**Figure 15:**
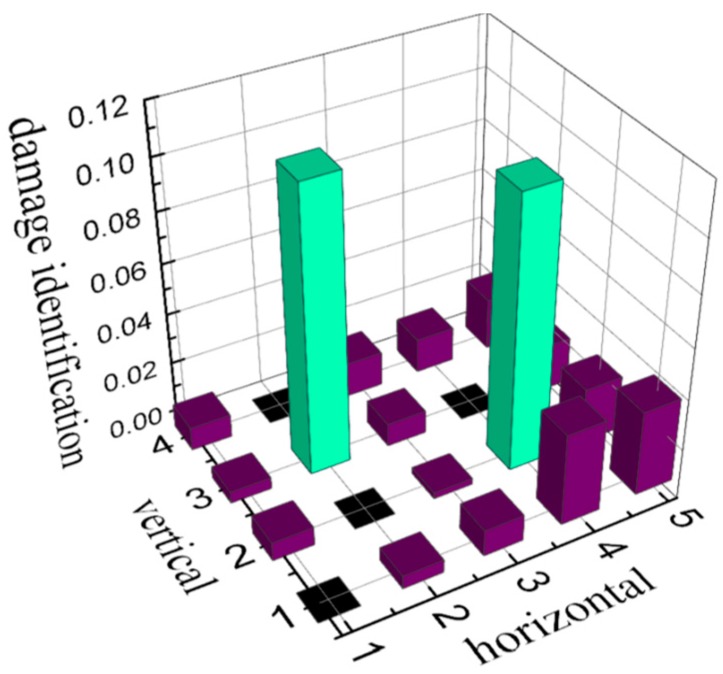
Damage identification factors.

The acceleration response and excitation data of the damaged tank were measured by the sensors that were placed in the middle of each substructure. The VDM and Equation (1) are employed to calculate the corresponding FRFs of each substructure with 0.9 kg virtual mass. Finally, the second natural frequencies are found by peak picking. When the frequencies of each substructure are listed in Table 5, it can be found that the frequencies of the damaged substructures *S*_3,2_ and *S*_2,4_ are obviously lower than those of the left and right substructures. According to Equation (2), the damage identification factors of each substructure are calculated and shown in Figure 15.

It can be clearly seen that the damage factors of substructures *S*_3,2_ and *S*_2,4_ are much higher than those of the other substructures, indicating that damage exists in substructures *S*_3,2_ and *S*_2,4_. Note that this result (detection and localization of the damages) is obtained without employing any FE model of the tank.

#### 3.4.3. Identification of Damage Degree

**Figure 16:**
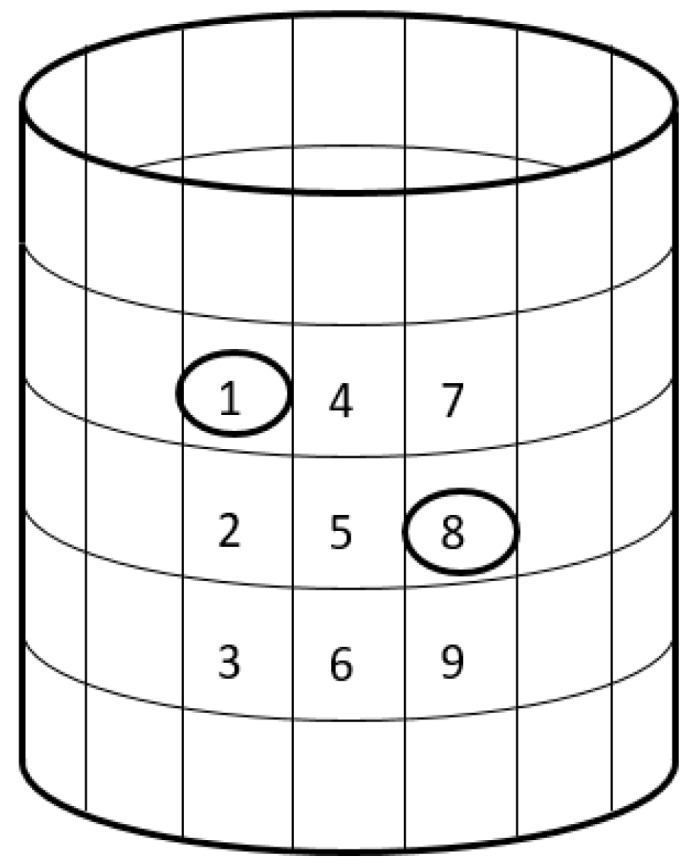
Schematic sketch of the substructures in the damage area.

Through an analysis of the damage factor in the previous section, the damage location, and a first rough estimation of the damage degrees are obtained. However, to achieve more accurate quantitative results, it is necessary to identify the damage factor of the structures employing the FE model. To facilitate optimization, the nine substructures of the damaged area found in the previous section are renumbered to 1–9, as shown in Figure 16.

**Figure 17:**
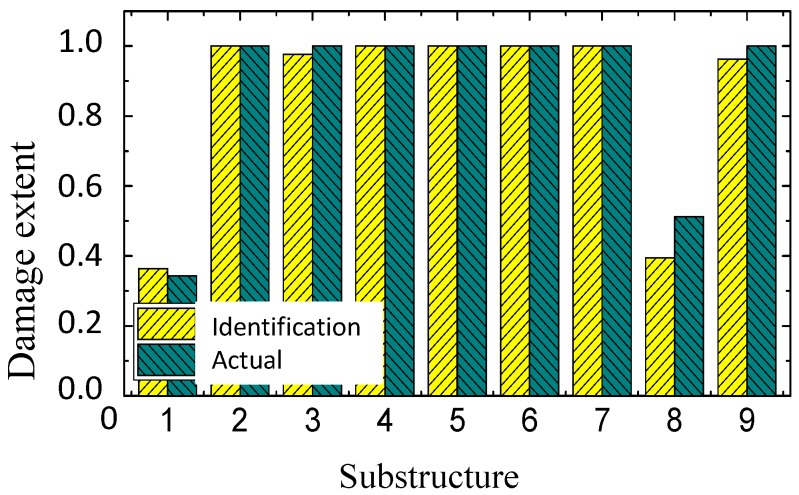
Identified damage.

**Figure 18:**
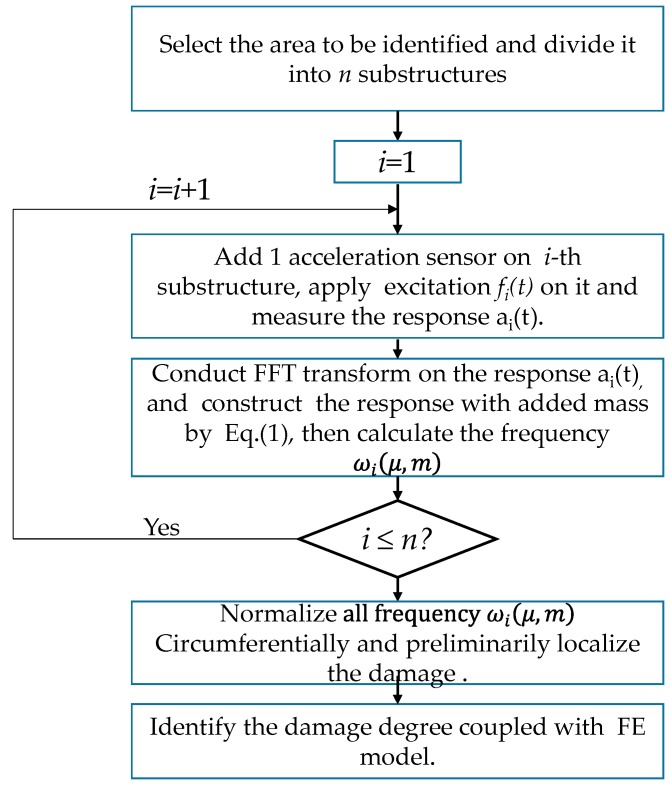
The flowchart of this proposed method.

In the previous sections, the natural frequencies are calculated after adding a 0.9 kg virtual mass to each substructure in the damage area. Using this result and the numerical FE model, the sensitivity optimization is carried out according to Equation (11), and the damage factors that were identified in multiple iterations are shown in Figure 17. The flowchart of the proposed technique is shown in Figure 18.

It can be found that the identified damage factors of substructures 1 and 8 are 0.3637 and 0.3947, respectively, while the thickness of steel plate of these two substructures is decreased to 0.7 and 0.8 of its original value, respectively. The difference is because the stiffness reduction ratio is not directly proportional to the thickness reduction ratio. According to the basic formula of the cross-section stiffness for a plate, the stiffness reduction ratio is close to the third power of the thickness reduction ratio.

## 4. Conclusions

Aiming at the characteristics of symmetric spatial geometry, the resulting dense low-order modes and insensitivity of global responses to local damages of tanks, a damage identification method by adding virtual masses is proposed. The validity of the method is verified by a comparison with a FE model of the tank, and it is then further validated experimentally using a lab-scale tank. The following conclusions are obtained:

(1) By adding virtual masses to the tank, the modal characteristics of the tank are changed. The frequency information with a high sensitivity to local damage can be obtained and the amount of test modal data can be increased.
(2) Based on the basic theory of the VDM, the frequency response of the tank with an arbitrary mass added is constructed by employing only a single pair of the excitation and acceleration responses of the original structure, which further increases the flexibility and applicability of the method.
(3) A set of frequency responses can be obtained by sequentially adding a virtual mass to each substructure of the tank structure. The damage location can be determined by comparing the selected natural frequencies of the substructures at the same height, without any FE model. Finally, the accurate damage factors of substructures can be quantitatively identified by a sensitivity-based optimization that employs a numerical FE model. The feasibility of the approach in practical engineering has been verified.

## Figures and Tables

**Table 1:** The first three natural frequencies/Hz.

Order	1	2	3
Frequency	330.71	526.86	739.38

**Table 2:** Comparison of the first three natural frequencies before and after stiffness correction/Hz.

Order of Frequency	1	2	3
Original	330.71	526.86	739.38
Modified	316.12	503.36	727.79
Experimental value	320.83	502.05	720.19
Error	1.47%	0.26%	1.06%

**Table 3:** Comparison of the first 3 natural frequencies after adding virtual and real masses/Hz.

Mass	Type	*S*_1,2_	*S*_2,2_	*S*_3,2_	*S*_4,2_
0.3 kg	VDM	478.76	470.83	468.76	458.56
	True	472.67	470.96	474.17	456.40
	Error	1.29%	0.03%	1.14%	0.47%
0.6 kg	VDM	413.62	439.66	454.45	448.59
	True	415.55	439.09	456.21	441.19
	Error	0.46%	0.13%	0.39%	1.68%
0.9 kg	VDM	384.45	410.05	448.20	443.42
	True	384.83	416.68	448.61	440.08
	Error	0.10%	1.59%	0.09%	0.74%

**Table 4:** Comparison of the two natural frequencies before and after damage/Hz.

Order of Frequency	1	2	3
Undamaged	320.83	502.05	720.19
Damaged	317.16	498.27	713.22
Difference	1.14%	0.75%	0.98%

**Table 5:** The second natural frequency for all substructures with an additional 0.9 kg virtual mass, as computed by the VDM for the tank after damage/Hz.

	*H*1	*H*2	*H*3	*H*4	*H*5
*V*4	434.29	438.41	432.48	432.75	429.94
*V*3	437.68	397.27	435.31	439.55	431.74
*V*2	408.87	411.08	410.60	367.25	402.53
*V*1	385.16	383.45	381.28	371.43	372.19
